# Human Small Intestinal Tissue Models to Assess Barrier Permeability: Comparative Analysis of Caco-2 Cells, Jejunal and Duodenal Enteroid-Derived Cells, and EpiIntestinal^TM^ Tissues in Membrane-Based Cultures with and Without Flow

**DOI:** 10.3390/bioengineering12080809

**Published:** 2025-07-28

**Authors:** Haley L. Moyer, Leoncio Vergara, Clifford Stephan, Courtney Sakolish, Hsing-Chieh Lin, Weihsueh A. Chiu, Remi Villenave, Philip Hewitt, Stephen S. Ferguson, Ivan Rusyn

**Affiliations:** 1 Department of Veterinary Physiology and Pharmacology, Texas A&M University, College Station, TX 77843, USA; hlm5009@tamu.edu (H.L.M.); csakolish@tamu.edu (C.S.); hclin@tamu.edu (H.-C.L.); wchiu@tamu.edu (W.A.C.); 2 Institute of Biosciences and Technology, Texas A&M University, Houston, TX 77030, USA; leovergara@tamu.edu (L.V.); cstephan@tamu.edu (C.S.); 3 Roche Pharma Research and Early Development, Roche Innovation Center Basel, F. Hoffmann-La Roche Ltd., 4070 Basel, Switzerland; remi.villenave@roche.com; 4 Merck KGaA, 64293 Darmstadt, Germany; philip.hewitt@merckgroup.com; 5 Division of Translational Toxicology, National Institute of Environmental Health Sciences, Research Triangle Park, NC 27709, USA; stephen.ferguson@nih.gov

**Keywords:** tissue chip, bioavailability, new approach methods

## Abstract

Accurate in vitro models of intestinal permeability are essential for predicting oral drug absorption. Standard models like Caco-2 cells have well-known limitations, including lack of segment-specific physiology, but are widely used. Emerging models such as organoid-derived monolayers and microphysiological systems (MPS) offer enhanced physiological relevance but require comparative validation. We performed a head-to-head evaluation of Caco-2 cells, human jejunal (J2) and duodenal (D109) enteroid-derived cells, and EpiIntestinal^TM^ tissues cultured on either static Transwell and flow-based MPS platforms. We assessed tissue morphology, barrier function (TEER, dextran leakage), and permeability of three model small molecules (caffeine, propranolol, and indomethacin), integrating the data into a physiologically based gut absorption model (PECAT) to predict human oral bioavailability. J2 and D109 cells demonstrated more physiologically relevant morphology and higher TEER than Caco-2 cells, while the EpiIntestinal^TM^ model exhibited thicker and more uneven tissue structures with lower TEER and higher passive permeability. MPS cultures offered modest improvements in epithelial architecture but introduced greater variability, especially with enteroid-derived cells. Predictions of human fraction absorbed (F_abs_) were most accurate when using static Caco-2 data with segment-specific corrections based on enteroid-derived values, highlighting the utility of combining traditional and advanced in vitro gut models to optimize predictive performance for F_abs_. While MPS and enteroid-based systems provide physiological advantages, standard static models remain robust and predictive when used with in silico modeling. Our findings support the need for further refinement of enteroid-MPS integration and advocate for standardized benchmarking across gut model systems to improve translational relevance in drug development and regulatory reviews.

## 1. Introduction

The state of science in cell-based models of gut permeability has advanced significantly in the past decade, largely driven by the need for more accurate predictions of oral drug absorption and metabolism, which are crucial for drug discovery and development for small molecules [1,2]. Many in silico and in vitro models have been developed to predict human drug absorption [3,4] or to better mimic the human intestinal epithelium in cell culture [5]. Both approaches are useful for the screening of drug candidates before absorption is measured in pre-clinical species; however, the lack of accuracy in these models is widely acknowledged [6,7] and a conservative approach is often followed to assume complete absorption in the gut [8].

The gastrointestinal tract is a complex tissue with anatomical, histological, and functional specializations along the continuum of its segments [9]. The gut is responsible for the effective and regulated nutrient transport, and the mechanisms of intestinal absorption of key nutrients vary among its segments. Because of the complexities in cell composition, topology of the surface, and expression differences in transporters, enzymes, microbiota, and immune mediators, both animal and cell-based models often fail to replicate human gastrointestinal physiology [10]. To address these limitations, a large number of in vitro models that use human cells have been developed in attempts to replicate intestinal epithelium properties that can be leveraged for absorption and metabolism studies [5]. The so-called 2D models (i.e., cell monolayers grown on permeable substrates), along with artificial membranes, are used most frequently due to their low cost and ease of use. For example, cell-free permeation models can be as simple as dialysis membranes or can be constructed from biomimetic materials such as phospholipids—examples include the Parallel Artificial Membrane Permeability Assay (PAMPA) or Phospholipid Vesicle-based Permeation Assay (PVPA) [11]. Cell-based models, particularly the Caco-2 cell line, are more commonly used even though they poorly mimic a sufficient breadth of active transport, intestinal metabolism, and complex paracellular transport due to overly tight junctions and lack of mucus production [12]. Other immortalized cell lines from the gut, such as TC-7, MDCK, HT-29, IEC, and HIEC, offer some advantages over Caco-2, such as faster growth, better mimicry of intestinal metabolism, mucus production, or paracellular transport; however, they each have limitations (e.g., non-human, de-differentiated cancer cell origins leading to incomplete differentiation, or lack of stable transporter expression) [5]. Several studies also showed that co-culture models, such as Caco-2 with HT29 or other cells, may improve physiological relevance such as mucus-secreting or immune-related functions [5]; however, it is to be established if these features improve human relevance for drug absorption. Organoids, derived from human intestinal stem cells, are the latest advancement in cell-based models for studies of the gut [13]. These enteroids form complex structures that include various intestinal cell types and reproduce the crypt–villus architecture; they demonstrate superior enzyme expression, mucus production, and transporter profiles compared to 2D models and can be cultured individually or placed on a membrane to study transport [14,15].

More recently, the innovation of in vitro gastrointestinal models has been focused on developing 3D models that address the needs of drug development [16]. These models aim to replicate the structural and functional features of the intestine more faithfully by recapitulating intestinal villi, allowing for proper tissue architecture and inclusion of multiple cell types, and introducing media flow and/or microbiota [17,18]. For example, villi-shaped scaffolds formed using hydrogels or nanofibers have been shown to provide a more realistic surface area and for improved modeling of drug permeability [19]. A number of membrane or gel-based microphysiological systems (MPS) have been developed and commercialized [20]; these allow for integration of dynamic flow conditions and mechanical forces [21,22,23]. Overall, MPS allow for improved control over cellular microenvironments, potentially improving drug transport and metabolism; however, they are also limited by technical complexity, higher costs, and challenges in standardization and scalability [24,25]. Indeed, standardization of the in vitro models is a critical need to improve confidence in their adoption by the end-users and regulators [26,27]. Despite the wide appreciation of the limitations with standard Caco-2 cultures on permeable membranes, this model has been widely used and is considered to be the workhorse for the initial evaluation of transcellular drug transport [12]. Relatively few cell-based models of intestinal transport have been rigorously evaluated for their robustness and reproducibility [28] or are offered as a service beyond the Caco-2 permeability assays. One alternative human intestinal model that includes multiple cell types (epithelial, fibroblasts, and endothelial) and has been extensively characterized in terms of human intestinal physiology and drug absorption is the EpiIntestinal^TM^ model, which is available commercially as pre-made tissue constructs on permeable membrane inserts in a multi-well format [29,30,31].

Few studies have compared the predictive performance of multiple complex intestinal models with varied cell types and inclusion of basolateral fluid flow. Several recent reports compared static and fluidic Caco-2 models and showed that the microfluidic MPS may have advantages in terms of human relevance for studies of peptides, such as insulin and octreotide [32], and small-molecule permeability data were more closely aligned to native tissues assessed in Ussing chambers [24]. However, the in vitro to in vivo correlation of permeability and fraction absorbed of the static model was similar to that in MPS, highlighting similar levels of predictivity but vastly different levels of complexity between these models [24]. Similarly, a comparative analysis of Caco-2 cells and human jejunal and duodenal enteroid-derived cells in gel- and membrane-based barrier models showed that that enteroid-derived cells cultured in gel-based MPS exhibited most physiological morphology, but that studies of drug permeability in this MPS were challenging [25]. These studies highlight the need for additional comparative analyses between MPS and more established in vitro models, a gap identified by many industry and government stakeholders [33,34]. Accordingly, this study aimed to evaluate several human small intestinal tissue models to assess barrier permeability. We conducted a comparative investigation of Caco-2 cells, jejunal and duodenal enteroid-derived cells, and EpiIntestinal^TM^ tissues in membrane-based cultures with and without fluid flow, which included cell morphology and barrier function evaluations across models, cells, and conditions. We also tested the permeability of three well-absorbed molecules and used these data as inputs into a physiologically based gut absorption model for the probabilistic prediction of human bioavailability.

## 2. Materials and Methods

### 2.1. Caco-2 Cell Culture and J2/D109 Enteroid Preparation

The human cell lines used in this study were Caco-2 cells, an epithelial cell line derived from the human colon (ATCC, Manassas, VA, USA; Cat. HTB-37), and human intestinal epithelial cells derived from enteroid cultures (J2 cells were derived from jejunum and D109 cells were derived from duodenum cells, 3D Organoids Core, Baylor College of Medicine, Houston, TX, USA). Caco-2 cells were grown at 37 °C, 5% CO_2_, in Eagle’s minimum essential medium (EMEM, ATCC, Cat. 30-2003) supplemented with 10% fetal bovine serum (FBS, ThermoFisher, Waltham, MA, USA, Cat. 16140071). To passage and obtain isolated cell suspensions, Caco-2 cells were treated with 0.05% Trypsin–EDTA (ThermoFisher, Cat. 25300054) for 5 min at 37 °C and resuspended in complete culture medium. J2 and D109 enteroids were obtained as 3D cultures in Matrigel and used within 3 days of delivery. To obtain plateable J2 and D109 cells, 3D cultures were first disaggregated with ice-cold 0.5 mM EDTA (Invitrogen, Grand Island, NY, USA, Cat. 15575-038) in sterile phosphate-buffered saline (PBS, Sigma-Aldrich, St Louis, MO, USA, Cat. D8537-500ML) and spun down at 4 °C to separate cells from Matrigel. The supernatant was removed, and the cell pellet was trypsinized (TrypLE^TM^, ThermoFisher, Cat. 12605010) for 30 min and 37 °C. Trypsin was inactivated by resuspending the cells in twice the volume of human enteroid growth medium (HEGM, Stem Cell Technologies, Vancouver, BC, Canada, Cat. 06010) supplemented with 10% FBS (ThermoFisher, Cat. 16140071). The disaggregation was completed by gently passing the cell suspension 50 times up and down through a P1000 pipette tip, followed by straining through a 40 µm membrane (pluriStrainer Mini, pluriSelect Life Sciences, Leipzig, Germany, Cat. 43-10040-40) to remove any remaining cell aggregates. After this, cells were spun down and supernatant removed, allowing cells to be resuspended in HEGM-Y medium (HEGM supplemented with 10 µM of the ROCK inhibitor Y27632, Sigma-Aldrich, St Louis, MO, USA, Cat. Y0503). For all these cell types, cell number and viability were evaluated using a TC20 automated cell counter (Bio-Rad Laboratories, Hercules, CA, USA, Cat. 1450103).

### 2.2. Cell Culture on Transwell^TM^ Inserts

Caco-2 cells were plated at 20,000 cells per insert on 6.4 mm Transwell^TM^ inserts (Corning #3470-Clear, 0.4 µm pore size; Sigma-Aldrich, Cat. CLS3470-48EA) placed in 24-well plates (Corning #3524) and cultured for up to 3 weeks before performing permeability assays as described elsewhere [35]. J2 and D109 cells were plated at ~109,000–180,000 and ~139,000–225,000 cells per insert, respectively, and cultured for 13 days prior to the permeability assay. The intestinal enteroid cell density was based on previous studies recommending ≥ 100,000 cells per 24-well (0.33 cm^2^) insert [36,37]. The inserts used for J2 and D109 cells were precoated with collagen IV (33 µg/mL, Sigma-Aldrich, Cat. C5533-5mg) prior to cell seeding. J2 and D109 cells were initially cultured in HEGM-Y and then switched to a differentiation medium (HEDM-Y) consisting of a 1:1 mixture of human organoid basal medium (Stem Cell Technologies, Cat. 100-0190) and DMEM/F12 (Stem Cell Technologies, Cat. 36254). For all cultures, media changes and TEER measurements were performed every 2–3 days.

Cells were either seeded conventionally on the upper surface of the insert membrane (top seeding, upright position) or on the underside of the insert membrane (bottom seeding, inverted orientation). For bottom seeding, sterile tweezers were used to place inserts upside down in a sterile pipette tip box, while for top seeding, inserts were kept in the original 24-well plates (Corning #3524). Prior to seeding, 100 µL of collagen IV (33 µg/mL, Sigma-Aldrich, Cat. C5533-5mg) was applied to the seeding surface of the inserts and incubated for 1.5 h at 37 °C. Then, the remainder of the coating solution was removed by tilting the plate and aspirating solution gently to avoid touching the bottom. Cells were prepared as described above and 100 µL of final cell suspension (2 × 10^5^, 1.09 × 10^6^–1.80 × 10^6^, and 1.38 × 10^6^–2.25 × 10^6^ cells/mL for Caco-2, J2, and D109 cells, respectively) was added to either the top or bottom of the Transwell^TM^ inserts. The seeded inserts were then carefully transferred to a cell culture incubator and maintained at 37 °C for 12 h to allow cells to attach. Following incubation, all inserts were carefully placed into 24-well plates, where 250 µL and 750 µL of maintenance medium was added to the apical and basal compartments, respectively. Following 2 days in the growth medium (HEGM-Y), TEER was assessed. If TEER reached a value greater than 100 Ohm×cm^2^, the inserts were transferred to MPS TC12 plates for fluidic culture or to 24-well plates for static culture, depending on the experimental setup.

### 2.3. PhysioMimix^®^ TC12 Plate Preparation

Before use, the CN Bio PhysioMimix^®^ system (CN Bio, Cambridge Science Park Milton Rd, Milton, Cambridge CB4 0WN, UK) was primed. The driver was placed into the culture hood and the MPS TC12 plate was removed from its packaging and inserted into the driver, closing the latch. To prime the TC12 plate, 750 µL of sterile HBSS was added to each well and the driver was attached to the docking station. The controller was set to “incubate” at 1 µL/s and maintained for at least 3 h to ensure priming was completed. After priming, the flow was stopped, and the driver was brought back to the biosafety cabinet. HBSS was removed, and 750 µL of media were added to each well. Inserts seeded with cells were then added to each TC12 well and the top compartment media were replaced with 250 µL of fresh media. The driver was placed back into the dock and the controller was set to “incubate” at 0.5 µL/s in the fluidic cultures. Media were changed each day to maintain cultures.

### 2.4. Cell Culture of Pre-Seeded EpiIntestinal^TM^ Tissues on Inserts

Upon receiving EpiIntestinal^TM^ tissues (SMI-100, kit of 24 pre-seeded inserts, MatTek, Ashland, MA, USA), inserts were set up on 12-well hang-top plates (HNG-TOP-12 plates, included as components of the SMI-100 kit) for culture under air–liquid interface (ALI) conditions, at 37 °C, 5% CO_2_. A total of 100 µL of medium was added to the top compartments and 5 mL were added to the bottom compartments. One day after receiving pre-seeded inserts, media were pre-warmed for chemical exposures and inserts were transferred to 24-well plates (Costar #3524). All endpoints (TEER, Dextran leakage, and chemical permeability) were collected from these 24-well plates, with 200 µL of pre-warmed media added to the apical side and 500 µL to the basal side of each insert.

### 2.5. Immunocytochemistry Staining and Optical Microscopy

For immunofluorescence staining of EpiIntestinal^TM^ inserts and cells grown on Transwell^TM^ inserts, culture media were removed from all top and bottom chambers and replaced with 4% paraformaldehyde (Electron Microscopy Sciences, Hatfield, PA, USA, Cat. 15710), followed by 15 min of incubation at room temperature and then washed with PBS. After fixation, cells were permeabilized with 0.3% Triton-X100 (ThermoFisher, Cat. A16046.AP), for 45 min at room temperature and then washed with PBS. Blocking was performed with PBS containing 2% BSA (Sigma-Aldrich, St Louis, MO, USA, Cat. A9418-50G), 2% goat serum (ThermoFisher, Cat. 16210064), and 0.1% Tween^®^ 20 (ThermoFisher, Cat. BP337-500) for 45 min at room temperature. Following blocking, actin staining was performed for two hours at room temperature using 5 U/mL of Alexa Fluor^TM^ 647 Phalloidin (ThermoFisher, Cat. A-22287). After rinsing, primary antibody incubation was performed overnight at 4 °C. Primary antibodies were used in the following combinations: rabbit polyclonal ZO-1 (1:250, ThermoFisher, Cat. 61-7300) and mouse monoclonal Ezrin (1:150, BD-Biosciences, Franklin Lakes, NJ, USA, Cat. 610602), or rabbit monoclonal Villin (1:100, Abcam, Cambridge, UK, Cat. ab130751). After primary antibody incubation, devices were rinsed with PBS and then incubated for 4 h at room temperature with a secondary antibody combination consisting of Goat Anti-Rabbit Alexa Fluor 488 (ThermoFisher, Cat. A-27034) and Goat Anti-Mouse Alexa Fluor 555 (ThermoFisher, Cat. A-21422) at 1:250 dilutions. After secondary antibody incubation and PBS rinsing, cells were counter stained for 10 min at room temperature with 1 µg/mL of DAPI (Invitrogen, Grand Island, NY, USA, Cat. D1306) and then washed with PBS. All incubation steps were performed in the dark. Following the staining, the Transwell^TM^ membranes were gently cut out from inserts and placed on glass slides with the cell side up. Mounting was performed using 50 µL of Slowfade Diamond Antifade Mountant (ThermoFisher, Cat. S36963) and 170 µm thick glass coverslips (18 × 18 mm), with clear nail polish sealing. Slides were then imaged with a spinning disk confocal microscope system consisting of a CSU-W1 spinning disk confocal scan head (Yokogawa, Tokyo, Japan) configured on a Nikon Ti2 inverted microscope.

### 2.6. Epithelial Permeability Test with Fluorescent Dextran

The integrity of the epithelial barriers formed by different cell types cultured in the Transwell^TM^ or EpiIntestinal^TM^ inserts was tested by measuring the permeability of 70 kDa TRITC-Dextran (Sigma-Aldrich, Cat. T1162-100MG). In MPS, the permeability from the cell-seeded chamber (donor) across the epithelium into the cell-free chamber (recipient) was measured using fluorescence imaging. The epithelial barrier integrity in EpiIntestinal^TM^ and Transwell^TM^ inserts was evaluated by measuring the leakage of 70 kDa TRITC-Dextran from the cell-seeded (donor) to the non-seeded (recipient) compartments in each well using fluorimetry. To perform the assay, the media from the selected inserts was removed (top and bottom) and the inserts were transferred to sterile, clear 24-well plates (assay plates), and Dextran loading solution (HBSS supplemented with 20 mM HEPES, 1.125 g/100 mL Glucose (HG-HBSS), and containing 0.5 mg/mL TRITC Dextran) was added to the donor (cell-seeded) compartment, while HG-HBSS was added to the recipient chamber. In addition to cell-seeded inserts, at least one cell-free insert was included to serve as a reference. The assay plate with the Dextran-loaded inserts was then transferred to a cell culture incubator and incubated in the dark for 2 h at 37 °C. At the end of the incubation period, the assay plates were removed from the incubator, the test inserts were transferred back to their respective culture plates, the loading solution was removed, and the cell line-specific culture medium was replaced after washing with HG-HBSS. Dextran fluorescence was measured directly in the assay plates using a plate reader with TRITC excitation and emission wavelengths, and unused wells containing fresh HG-HBSS were used as blanks. The relative leakage of Dextran was quantified by expressing the individual well measurements as percentage of the cell-free control wells (no epithelial barrier).

### 2.7. Trans-Epithelial Electrical Resistance (TEER) Measurements

TEER measurements were performed for all seeding conditions using an EVOM2 Epithelial Volt/Ohm Meter (World Precision Instruments, Sarasota, FL, USA) equipped with a 4 mm chopstick-style electrode (STX2, World Precision Instruments). The plates were transferred from the incubator to the biosafety cabinet and allowed to equilibrate at room temperature for at least 15 min prior to testing. To perform the measurements, the terminals of the chopstick electrode were immersed in 70% isopropyl alcohol and washed with sterile HBSS solution; the resistance in Ohms was measured by inserting the electrode vertically into each well with the long arm in the bottom compartment and the short arm in the top compartment. For EpiIntestinal^TM^ tissues, inserts were transferred to a 24-well plate containing 500 μL media, and 200 μL media were added to the top. TEER readings were measured as described above. An insert without cells was used as a reference (cell-free resistance). For analysis, reference values were subtracted from the experimental wells and the results multiplied by the insert area (0.33 cm^2^) for Transwell inserts and 0.6 cm^2^ for EpiIntestinal^TM^ inserts. The results are expressed as Ohms×cm^2^.

### 2.8. Chemical Treatments

To evaluate chemical transport across the cell barrier, caffeine, propranolol, or indomethacin, diluted in cell culture media as indicated in Section 3, were added to either the cell-seeded (apical) or non-seeded (basolateral) compartment of each model. Chemicals were purchased from Sigma Aldrich: caffeine (Cat no. 56396), propranolol (Cat no. PHR1308-1G), and indomethacin (Cat no. PHR1247-500MG). Untreated cell culture medium was added to the opposite channel. Chemical transport was evaluated over 100 min to understand cell-mediated transport. Media were collected and stored at −80 °C for later analysis.

### 2.9. Analytical Chemistry Methods

Media samples that were collected during the experiments were stored at −80 °C until extraction with a liquid–liquid protein precipitation method. The following internal standards were obtained from Sigma-Aldrich: C_13_-caffeine (1 µM; Cat no. C-082-1ML), nadolol (internal standard for propranolol, 1 µM; Cat no. N1892-1G), and naproxen (internal standard for indomethacin, 5 µM; Cat no. N8280-5G). Briefly, 100 µL of ice-cold acetonitrile (ThermoFisher, Cat no. A998-4) containing internal standards was added to 50 µL of each media sample and briefly vortexed to precipitate out proteins. The supernatant was then evaporated using a Savant^TM^ SpeedVac^TM^ (ThermoFisher, Cat no. SPD1010) and samples were reconstituted with mobile phase A (HPLC-grade water containing 0.1% Formic Acid; Sigma-Aldrich: Cat no. 576913) before being transferred to LC-MS vials (Ibis Scientific, Tampa, FL, USA, Cat no. 4400-FIV2W). After extraction, the samples were stored at −20 °C until instrumental analysis.

Extracted caffeine, propranolol, and indomethacin samples (10 µL, 3 µL, or 5 µL respectively) were injected onto a ZORBAX SSHD Eclipse Plus C18 column (3.0 × 50 mm, 1.8 μm, Cat. 959757-302, Agilent, Santa Clara, CA, USA) with a guard column (2.1 × 5 mm, 1.8 μm, Cat. 821725-901, Agilent) at 40 °C using a 1290 Infinity II LC (Agilent) and analyzed using a triple quad mass spectrometer (Agilent 6470A). All analyses were performed using positive electrospray ionization. Gas Temp, Sheath Gas Temp, and nebulizer pressure were set to 350 °C, 300 °C, and 35 psi for the analysis of caffeine and propranolol. Parameters for the analysis of indomethacin were 290 °C, 350 °C, and 40 psi. Binary Pump composition for the analysis of caffeine and propranolol began with 90% mobile phase A and 10% mobile phase B (acetonitrile with 0.1% formic acid; ThermoFisher; Cat no. A998-4), then was increased to 80% mobile phase B at minute 3. Mobile phase B was increased to 95% at minute 4, then returned to 10% mobile phase B at minute 5. Sample run time ended after 7 min. Binary pump gradient for indomethacin began with 90% mobile phase A and 10% mobile phase B, then was increased to 80% phase B at minute 3. Mobile phase B was then increased to 90% at minute 4. The gradient returned to 10% B at minute 6 until the run ended at minute 8. The limits of detection for caffeine, propranolol, and indomethacin were 0.0015 µM, 0.0051 µM, and 0.03 µM, respectively. The lower limits of quantification were 0.0015 µM, 0.015 µM, and 0.03 µM, respectively. The limits of quantification and detection were based on linear (r^2^ > 0.97) calibration curves.

Calculations of apparent permeability ‘*P*_app_’ (Equation (1)) were performed over 100 min, where ‘dC’ is the change in concentration in moles in the recipient compartment, and ‘dT’ is the change in time in seconds. ‘A’ represents the area over which the chemical has to cross in and C_0_ is the initial concentration in the donor compartment. Data are presented as ×10^−6^ cm/s.

(1) $$P_{\mathrm{app}}=\frac{\left(\frac{dC}{dT}\right)\times V_{R}}{\;(A\times C_{0})\;}$$

### 2.10. PECAT Modeling

A previously developed physiologically based probabilistic environmental compartmental absorption and transit (PECAT) model [8] was employed to assess whether using segment-specific apparent permeability (*P*_app_) from different gastrointestinal segments and models could enhance the accuracy of gut absorption fraction (F_abs_) predictions. It was previously reported [8] that the best performance for predicting F_abs_ using the PECAT model was achieved when Caco-2 *P*_app_ values were used as input, which were first converted to in vivo jejunum permeability using in vitro-to-in vivo correction factors and then adjusted with segment-specific ratios to estimate segment-specific permeability. Therefore, the following F_abs_ predictions primarily relied on this approach.

In this study, chemical- and segment-specific permeability data were measured in J2, D109, and Caco-2 cells in the PhysioMimix^®^ TC12 plate, as well as permeability data measured in ileum cells using EpiIntestinal^TM^ inserts (only permeability tested in the A to B assay were used), were applied as input permeability. These values were then used to calculate chemical- and segment-specific ratios to jejunum permeability. Five different simulation approaches were designed to compare the predictive performance of F_abs_. [i] Using Caco-2 P_app_ from PhysioMimix^®^ TC12 plate with the distributions of in vitro-to-in vivo correction ratio and segment-specific ratios from [8]. [ii] Using Caco-2 *P*_app_ from PhysioMimix^®^ TC12 plate with the distributions of in vitro-to-in vivo correction ratio and segment-specific ratios derived from experimental *P*_app_ values of J2, D109, and Caco-2 cells. [iii] Using J2 *P*_app_ from PhysioMimix^®^ TC12 plate alone, with default segment-specific ratio distributions. [iv] Using J2 *P*_app_ PhysioMimix^®^ TC12 plate alone, with segment-specific ratios derived from the experimental *P*_app_ values of J2, D109, and Caco-2 cells. [v] Using ileum *P*_app_ from EpiIntestinal^TM^ inserts and assuming this *P*_app_ to be equal to jejunum permeability, with default segment-specific ratio distributions.

Approaches [i] and [ii] were based on the previous findings [8], and aimed to evaluate the impact of using chemical-specific J2 and D109 permeability data to obtain the segment ratios for improving F_abs_ predictions. Approaches [iii] and [iv] assessed the impact of replacing Caco-2 with J2 in the PhysioMimix^®^ TC12 plate for estimating jejunum permeability, without considering the in vitro-to-in vivo correction ratio. Approach [v] primarily aimed to evaluate how different MPS models affect F_abs_ predictions, which compared to Approach [iii].

All PECAT model simulations, which incorporated distributions of ratios, were performed using the Monte Carlo algorithm via the GNU MCSim v.6.1.0 within the R environment. Observed human F_abs_ values for the three compounds were collected from the literature and used to compare against model predictions to evaluate performance. The mean observed human F_abs_ for propranolol was 96.3%, while for the other two chemicals, the values were 100% [8].

### 2.11. Statistical Analyses

Data analysis was performed using GraphPad Prism (version 10.2.0, San Diego, CA, USA). All error bars shown represent the standard deviation and sample numbers are indicated in the figure legends. Data were analyzed using *t*-tests or 2-way ANOVA with post hoc testing as indicated in the figure legends.

## 3. Results

**Figure 1:**
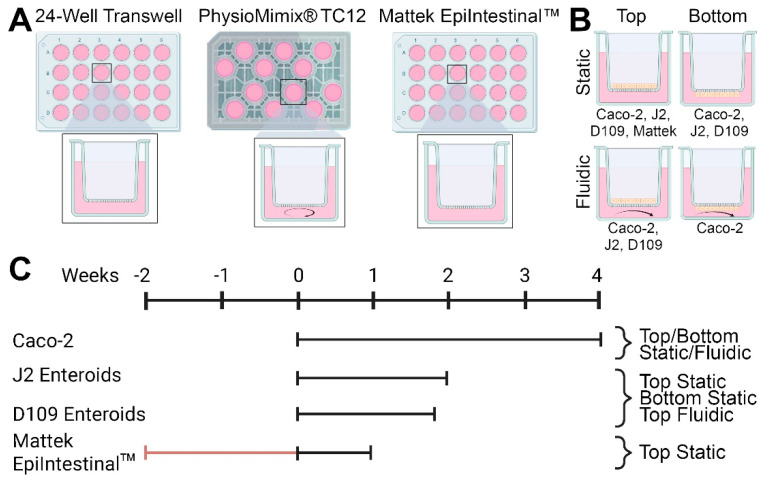
General designs for the studies reported in this study. (**A**) Three model systems were evaluated—24-well Transwell inserts, PhysioMimix^®^ TC12 microphysiological system, and Mattek EpiIntestinal^TM^ model. (**B**) Four human cell types (Caco-2 cell line, J2 and D109 human enteroids, and primary small intestinal epithelial cells) were cultured in 4 different conditions. Cells were cultured either inside the transwells (“top”) or on the outside of the membrane (“bottom”) and were cultured undisturbed (“static”) or with recirculating media flow (“fluidic”). Cell types used in each condition are indicated. (**C**) Experimental timelines for each cell type and condition. Day 0 corresponded to the day when the experiments were started in the laboratory—either cells were seeded into devices as detailed in Section 2 (for Caco-2 cell line, J2 and D109 human enteroids) or received from the vendor (Mattek EpiIntestinal^TM^).

This study conducted a head-to-head comparison of several in vitro models of intestinal permeability (Figure 1). We tested static and fluidic Transwell and static EpiIntestinal models (Figure 1A). Cells (Caco-2, human jejunal and duodenal enteroids, or EpiIntestinal multi-cellular tissues) were seeded either on top or bottom of membrane inserts, depending on the cell type and model (Figure 1B). Study duration, from 1 to 4 weeks, also varied depending on the cell type and model (Figure 1C). Detailed study protocols and all images and data from these experiments are available through the EveAnalytics database (Table S1).

**Figure 2:**
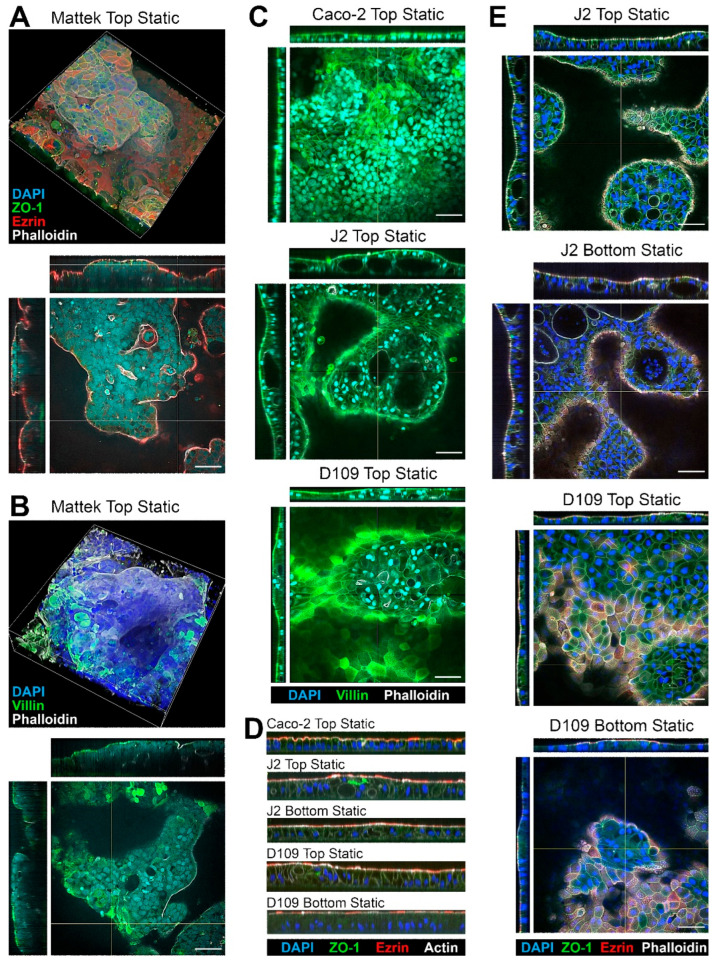
Representative images of the intestinal tissues formed with different cells and culture conditions. (**A**,**B**) Examples of the Mattek EpiIntestinal^TM^ model. Staining for nuclei (DAPI, blue) and apical (Ezrin, red; Phalloidin, white; and Villin, green) and lateral (ZO-1, green) membrane markers of gut epithelial cells is shown. Both top (single plane) and side cross-sections (yellow lines mark the location) are shown. Images are shown at 40× magnification and the scale bars are 50 μm (all images shown in the entire figure are scaled the same). (**C**) Examples of Caco-2 cells, and J2 or D109 enteroids. (**D**) Representative sagittal sections showing the differences in the thickness of the epithelial layer for different cell types and culture conditions. See Figure 3 for quantitative information on the epithelial layer in each model. (**E**) Comparison of tissue morphology between J2 and D109 enteroids cultured at the top or bottom of a transwell without flow.

All cells tested in this study formed confluent layers on membrane inserts; tissue morphology and expression of key makers were evaluated using fluorescence microscopy (Figure 2). Because of the highly variable thickness of the cell layers, only qualitative analysis was possible with respect to the expression of typical intestinal epithelium markers. Representative 3D images of EpiIntestinal tissues are shown in Figure 2A,B.

These tissues came pre-seeded inside a permeable support insert that could not be placed into the PhysioMimix^®^ TC12 plates; therefore, they were only tested in the “top static” condition. Complex variable-thickness cell layers were observed on each insert. Robust staining for ZO-1 showed a typical intestinal epithelium pattern along the cell–cell contact sites, indicating presence of the tight junctions [38]. Staining for Ezrin, an apical membrane—“brush border”—marker [39] showed a uniform apical band along the epithelial continuum indicating the presence of villous enterocytes. Villin staining showed a similar pattern but also visualized the areas where denser microvilli were present [40]. Finally, Phalloidin, a fluorescent marker for actin filaments [41], also showed a strong apical signal due to presence of actin in the microvilli.

Caco-2 cells, as well as J2 and D109 enteroids, were compared in the same “top static” configuration (Figure 2C). Caco-2 cells showed a more uniform cell monolayer as compared to either J2 or D109 enteroids. The latter two formed domed structures, occasionally with cysts in some thicker areas of the epithelium (see J2 Top Static cross-sections). Similar to EpiIntestinal^TM^ tissues, robust staining for villin was observed; however, villin was enriched in the brush border in J2 and D109 cultures with J2 signal being far more concentrated as an apical band because of the denser villi in human jejunum, as compared to duodenum [42]. Similar patterns of staining were also observed on the sagittal sections (Figure 2D)—dense brush border staining for Ezrin and Actin and taller columnar epithelium in J2 and D109 enteroids as compared to Caco-2 cells. Figure 2E compares top vs. bottom seeded J2 and D109 cells under static culture conditions. In both configurations, J2 cells formed highly variable cell layers with cysts; intense brush border staining was evident in all cases, regardless of the top or bottom seeding. The bottom-seeded fluidic images are not shown for the J2 and D109 enteroids because these cells did not form adhered tissues in this condition—even a very low flow of 0.5 mL/min was sufficient to disrupt tissue attachment to the insert and preclude studies with these enteroids in fluidic conditions.

**Figure 3:**
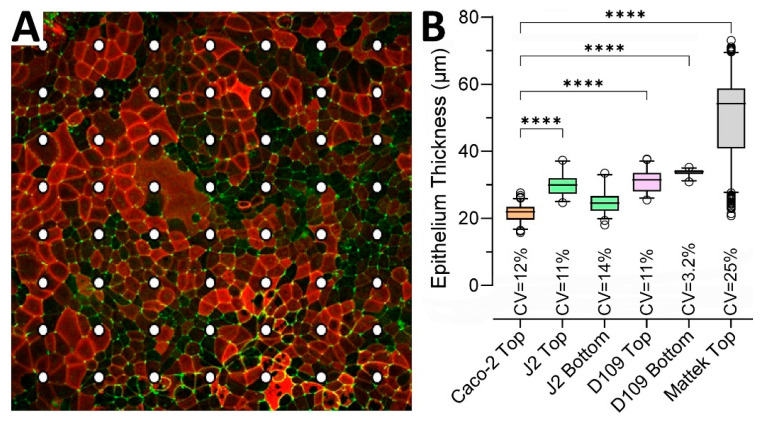
Intestinal tissue thickness for different cells and seeding conditions. (**A**) Examples of the measurement grid applied to evaluate epithelium thickness in cultures of different cells and seeding conditions. Shown is an example from Caco-2 cells seeded inside a static transwell. The image is a maximum intensity projection of a z-stack with staining for apical (Ezrin, red) and lateral (ZO-1, green) membrane markers. Dots indicate the grid where epithelial thickness was measured for each model at 40× magnification. (**B**) Data from different experiments were combined as indicated and are shown as box and whisker plots (horizontal line is median, box is inter-quartile range, whiskers are 5–95 percentile range, and outliers are hollow circles). All pairwise comparisons (except for J2 Top vs. D109 Top) were significant (*p* < 0.05) based on a one-way ANOVA analysis followed by Tukey’s multiple comparison test. Shown are pairwise comparisons (**** indicates *p* < 0.0001) for each group against Caco-2 Top as a reference. Also shown are the coefficient of variability (CV) values for each group.

To quantify epithelial thickness in each condition, we examined representative inserts for each of the conditions shown in Figure 3 using a uniform grid of cross-sections. Figure 3A shows an example of how the points of measurement were distributed on each insert—the illustration shows Caco-2 cells that form a largely uniform monolayer with robust ZO-1 staining but uneven staining for ezrin. Figure 3B shows quantitative analysis results confirming that the Caco-2 monolayer was the thinnest (geometric mean and SD of 21.4 ± 1.1 μm) among all conditions and significantly different from all conditions, except for the second thinnest J2 bottom-seeded cells (24.5 ± 1.2 μm). EpiIntestinal^TM^ tissues were twice as thick as other tissues (49.0 ± 1.3 μm). Monolayer thickness was also most variable for EpiIntestinal^TM^ tissues, with a coefficient of variability (CV) of 25%.

**Figure 4:**
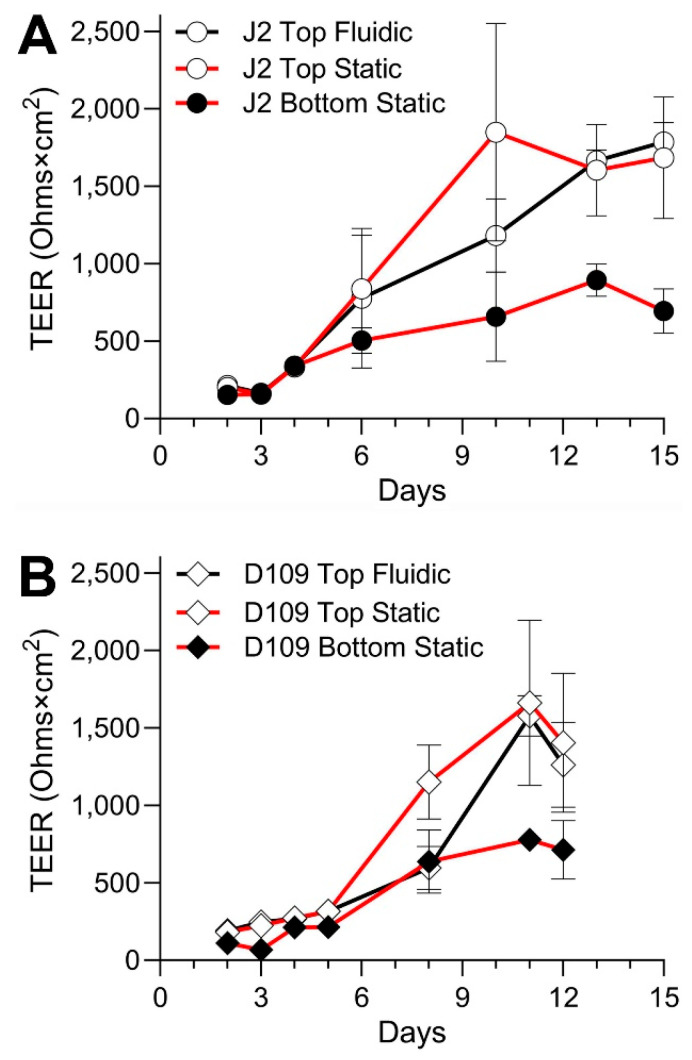
Time course of the barrier formation in the experiments with J2 and D109 enteroids. (**A**) Data for J2 enteroids are shown for three separate culture conditions as indicated in the legend. Data from different experiments were combined and plotted are mean ± standard deviation values for the measurements taken on each experimental day. Cells were not treated on any of the days shown. (**B**) Similar data for D109 enteroids. The experiments were stopped 3 days earlier than those with J2 enteroids.

While EpiIntestinal^TM^ tissues arrive pre-seeded and immediately ready for testing, other models take time to establish robust monolayers that can be evaluated non-invasively using TEER measurements. Figure 4 shows the time course of TEER values in experiments with J2 and D109 enteroids. Both cell types reached their peak TEER around day 11–12 in culture; however, D109 cells showed a consistent trend of decreasing TEER almost immediately after the peak was reached, while J2 cells exhibited a more prolonged plateau of several days. It is also noteworthy that both cell types cultured on the bottom side of the insert achieved only about one-half of the TEER reached when cells were inside the insert. Also, in both cases, the top-seeded cells achieved their highest TEER slower, albeit to the same level, when they were cultured in PhysioMimix^®^ TC12 with the media flow on the opposite side of the insert. Overall, these data show that D109 cells were used for chemical permeability testing several days sooner than J2 cells because of the deteriorating TEER past day 11 vs. day 13 in culture, respectively.

**Figure 5:**
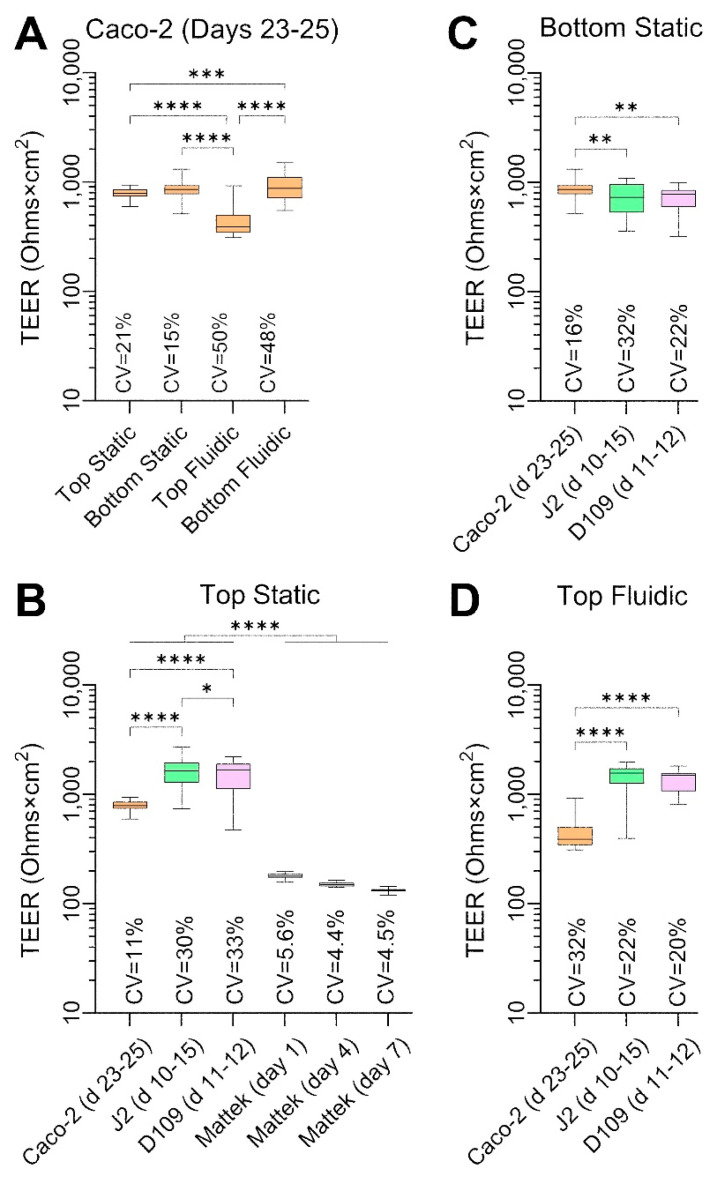
Comparative analysis of TEER values in each cell type and culture condition. Data are shown for untreated cells for the indicated experimental days. Data from different experiments were combined as indicated and are shown as box and whisker plots (horizontal line is median, box is inter-quartile range, and whiskers are min–max values). Asterisks indicate pairwise significance from a one-way ANOVA analysis followed by Tukey’s multiple comparisons test (*, *p* < 0.05; **, *p* < 0.01; ***, *p* < 0.001; and ****, *p* < 0.0001). Also shown are the coefficient of variability (CV) values for each group. (**A**) Data for Caco-2 cells for four separate culture conditions on days 23–25. (**B**) Data for 4 cell types that were cultured inside transwells under static condition. For Mattek EpiIntestinal^TM^ model, data are shown for 3 different time points on the same plate. (**C**,**D**) Data for Caco-2 cells and J2 and D109 enteroids that were cultured on the outside of transwells under static condition (**C**) or inside transwells under flow in the outside chamber (**D**).

A comparison of TEER values among cell types and culture conditions at their peak level is shown in Figure 5. Caco-2 cells cultured with or without flow and on different sides of the semi-permeable insert membrane showed a very high TEER (Figure 5A), albeit it was significantly lower in the top fluidic configuration. The variability in TEER, as assessed by CV, was close to 50% for PhysioMimix^®^ TC12 experiments regardless of whether the cells were in the direct path of the flow (bottom seeded) or not (top seeded); this was 2–3 times higher than CVs for static conditions. In the top static condition (Figure 5B), we could compare all cell types and EpiIntestinal^TM^ tissues. J2 and D109 enteroids had an even greater TEER than that for Caco-2 cells, at or above 1500 Ohms×cm^2^ and with greater CVs. By contrast, EpiIntestinal^TM^ tissues had a TEER between 100 and 200 Ohms×cm^2^ with very low CVs, consistent with previous publications on this model [29]. The EpiIntestinal^TM^ tissues exhibited a gradual decrease in TEER over 1 week in culture. In a bottom static condition (Figure 5C), J2 and D109 TEER was significantly lower than that in similarly cultured Caco-2 cells. In a top fluidic condition (Figure 5D), the trends were reversed.

**Figure 6:**
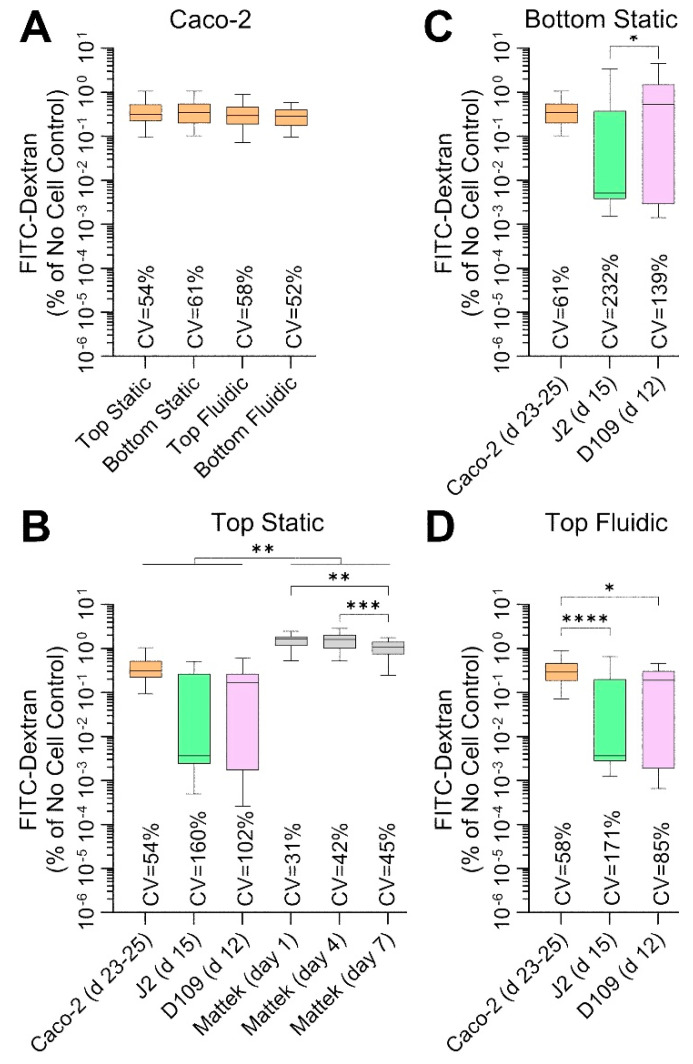
Comparative analysis of 70 kDa FITC-Dextran permeability in each cell type and culture condition. Data are shown as percent permeability over 100 min as compared to identical experimental condition without cells. Data from different experiments were combined as indicated and shown are box and whisker plots (horizontal line is median, box is inter-quartile range, and whiskers are min-max values). Asterisks indicate pairwise significance from a one-way ANOVA analysis followed by Tukey’s multiple comparisons test (*, *p* < 0.05; **, *p* < 0.01; ***, *p* < 0.001; and ****, *p* < 0.0001). Also shown are the coefficient of variability (CV) values for each group. (**A**) Data for Caco-2 cells for four separate culture conditions on days 23–25. (**B**) Data for 4 cell types that were cultured inside transwells under static condition. For Mattek EpiIntestinal^TM^ model, data are shown for 3 different time points on the same plate; days are indicating time periods after receipt of the tissue constructs (see Figure 1). (**C**,**D**) Data for Caco-2 cells and J2 and D109 enteroids that were cultured on the outside of transwells under static condition (**C**) or inside transwells under flow in the outside chamber (**D**).

Another measure of barrier integrity in studies of intestinal permeability is the ability of the fluorescently labelled molecules to penetrate across the cell layer on the perforated insert. Figure 6 shows the results of these studies—these measurements were collected at the same times and conditions as TEER measurements (Figure 5). Caco-2 cells showed very low permeability—less than 0.5% of 70 kDa Dextran crossed the barrier over 100 min—across all conditions (Figure 6A), albeit these data were highly variable with CVs exceeding 50%. Permeability was even lower (around 0.15%) for J2 and D109 enteroids in all conditions, but the data were highly variable with CVs between 85 and 232% (Figure 6B–D). By contrast, EpiIntestinal^TM^ tissues were most ‘leaky’ with permeability ~1.5% and variability comparable to that of Caco-2 cells (Figure 6B). Overall, the Dextran permeability data are highly concordant with TEER measurements, as expected, but the variability in TEER data was far lower than that for Dextran fluorescence.

**Figure 7:**
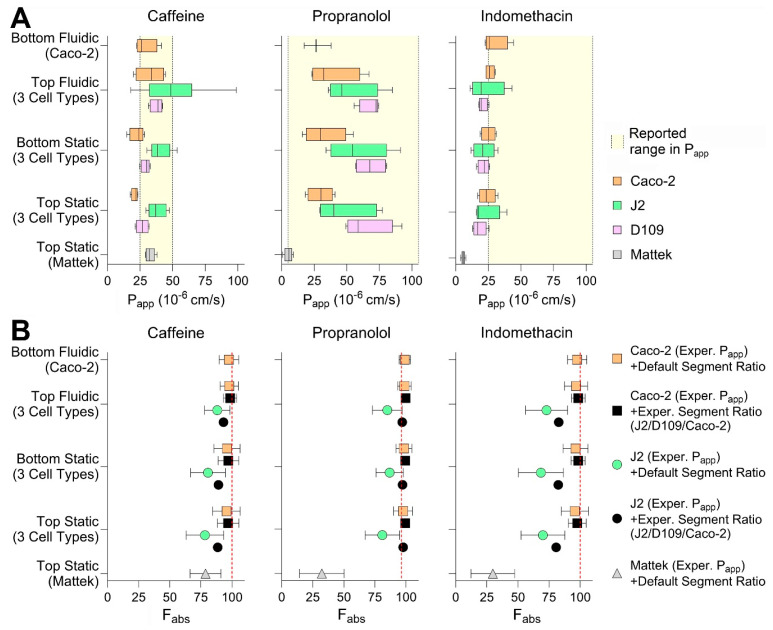
Chemical permeability in different intestinal tissues in vitro. (**A**) Permeability (P_app_) in different cell types and culture conditions at 100 min of exposure. Caffeine, propranolol, and indomethacin were tested at 100 μM, 10 μM, or 200 μM (respectively) in all conditions, except for Mattek EpiIntestinal^TM^ where they were tested at 10 μM, 1 μM, or 20 μM. Data are shown as box and whisker plots (vertical line is median, box is inter-quartile range, and whiskers are min-max values). Shaded regions indicate the range of previously published P_app_ values in Caco-2 transwell studies. (**B**) Predictions of the fraction absorbed (F_abs_) by using various combinations of permeability of Caco-2 and J2 cells measured by different models tested in this paper, in vitro-to-in vivo correction and GI segment-specific ratios using the PECAT model [8]. Vertical dashed lines represent the observed human F_abs_.

Finally, we tested three small molecules—caffeine, propranolol, and indomethacin—all known to be completely (or near completely for propranolol) absorbed when administered via the oral route in each of the conditions. We aimed to evaluate whether drug- and segment-specific data from the in vitro intestinal models tested in this paper can be used to increase accuracy and precision of in silico predictions of gut absorption [8]. A physiologically based gut absorption model was parameterized with the data obtained from each cell type and model, and the results were compared among the conditions tested in this paper and with the human gut absorption data (Figure 7). Specifically, we derived the permeability values for each of the tested chemicals in each experimental condition (i.e., cell types, model, seeding condition, and presence or absence of media flow; Figure 7A). We found that, most of the time, the *P*_app_ values derived experimentally were within the range of previously reported values (Table S2). Generally, Caco-2-derived values were the lowest as compared to J2 and D109 enteroids; however, the *P*_app_ values from EpiIntestinal^TM^ tissues were the lowest for propranolol and indomethacin. Still, the seeding condition and presence of flow did not make a difference in *P*_app_ values for tested compounds. Experimentally derived permeability values and gut segment ratios were then used in the PECAT model [8] to predict human absorption (fraction absorbed, *F*_abs_). The predicted *F*_abs_ for each of the chemicals and when using five different scenarios (see Section 2.10) are illustrated in Figure 7B. Our results indicate that the experimental data from top seeded static Transwell inserts—Caco-2 *P*_app_ and segment-specific (J2 vs. D109 vs. Caco-2) ratios—yielded the most accurate and precise predictions of *F*_abs_ for the three compounds tested. The use of these data was notably superior compared to predictions based on J2 permeability values, which severely underestimated *F*_abs_. By contrast, predictions using the EpiIntestinal^TM^ model underestimated *F*_abs_ when based on ileum *P*_app_ values and default segment-specific ratio distributions.

## 4. Discussion

In vitro modeling of intestinal absorption and metabolism, or general toxicity, is a particularly challenging area in drug development. On the one hand, the gastrointestinal system is a highly metabolically active tissue with unique anatomical and morphological features (length, surface area, and segment-specific gene expression and function), and it is also the site of administration of most drugs and chemicals that come into the human body [9,43]. On the other hand, important species differences exist in the manifestations of adverse effects of drugs on the gastrointestinal system [44]. In addition to predicting intestine toxicities that are common dose-limiting adverse events in drug development [16,45], or developing new models for studies of debilitating diseases of the gut [46], a persistent need exists to characterize drug absorption before animal or human studies are initiated [5]. There are many possible choices for addressing the latter challenge; the difficulty is in determining their robustness, reproducibility, domain or applicability, throughput, and ultimately the overall ‘value proposition’ for a specific context of use—estimating the fraction absorbed in humans after oral administration. While a conservative assumption can be made that oral absorption is complete, the precision in the estimate of bioavailability is often consequential for dose and candidate selection [47].

Given the need to better understand how complex models compare against more simple and traditional in vitro models, our study provides a head-to-head evaluation of multiple human small intestinal tissue models such as Caco-2 cells, jejunal and duodenal enteroid-derived monolayers, and commercially available EpiIntestinal^TM^ tissues. These models were compared across standard membrane-based and an MPS platform, with and without flow. The comparative design of our experiments, combined with the direct evaluation of epithelial morphology, barrier function, and chemical permeability, contributes significantly to the field of intestinal in vitro modeling by demonstrating how the reproducibility and robustness of the gut barrier function and drug absorption can be performed.

Our findings confirm and extend previous work on the limitations of Caco-2 monolayers [12,35,48] by demonstrating marked differences in epithelial thickness, barrier permeability (TEER and Dextran leakage), and segment-specific marker expression among the models and cell types tested. Importantly, this study is among the first to compare permeability data across cell lines and primary enteroid-derived cultures under static and fluidic conditions, and to incorporate these data into a physiologically based absorption model (PECAT) for the in silico prediction of oral drug bioavailability. By integrating TEER, Dextran permeability, and small-molecule *P*_app_ values into a segment-informed framework, we demonstrated that data from jejunal (J2) and duodenal (D109) enteroids are more informative with respect to in vivo segmental physiology and drug permeability compared to Caco-2 cells alone. The observed morphological features, including taller columnar epithelium, dense brush border (villin, ezrin, and phalloidin), and segmental differences, mirror in vivo intestinal physiology, affirming their potential for both mechanistic studies and applied pharmaceutical research. This offers a compelling rationale for incorporating enteroid-derived models into future predictive drug transport workflows [49,50]. Furthermore, enhanced cell types and modeling aspects of 3D tissue architectures are reported to confer enhanced drug metabolism proficiency [51]. The EpiIntestinal^TM^ model showed high reproducibility and physiological tissue morphology but also demonstrated the lowest TEER and the highest passive permeability among models, suggesting a greater paracellular component or incomplete barrier integrity in this setting. These insights may guide the selection of in vitro models for different study goals (e.g., toxicity vs. absorption). Notably, despite the theoretical advantages of MPS or EpiIntestinal^TM^ model, our PECAT-based simulations showed that *P*_app_ values derived from standard static cultures (especially top-seeded Transwell inserts) were equally or more effective at predicting human oral bioavailability for the tested compounds. This suggests that, while MPS or more complex intestinal tissues may offer structural and physiological realism, their added value for parent drug permeability prediction may not yet outweigh the practicality, robustness, and translatability of conventional models in all use cases.

It is also possible to place our study into the context of recent work by others who explored new insights into the value of MPS versus standard assays. The implementation of a flow-based MPS (PhysioMimix^®^ TC12) in our study highlighted both the promise and the current limitations of these advanced systems. While the MPS enabled dynamic culture environments and modestly affected TEER in some configurations, it also introduced increased variability and presented technical challenges, particularly with enteroid-derived cultures, which detached under even minimal flow conditions. These observations align with prior reports that underscore challenges in scaling and standardizing MPS for high-throughput or regulatory adoption [26,33,52]. For example, two recent studies that used another flow-based MPS model—the Emulate system—provided somewhat divergent conclusions with respect to the added value offered by the MPS [24,32]. One study [24] evaluated 19 representative small molecules using both traditional static Transwell and fluidic Emulate models seeded with Caco-2 cells. Both viability and absorption were assessed, and it was found that both models provided highly variable data for each tested compound. Still, both systems demonstrated similar predictive strength with r^2^ values ranging from 0.4 to 0.8. The authors did find that the MPS model’s permeability data more closely matched native human tissue profiles from Ussing chamber data; this led to a conclusion that a commercially-available MPS model can be used for predicting oral drug absorption, yet the added cost and complexity need to be taken into account. A companion study [32] that compared the same models with respect to their utility for studies of intestinal permeation enhancers and oral peptide delivery concluded that the MPS was more useful than typical in vitro models for that specific context of use.

One other notable observation from our data is the comparison between cell types and models with respect to the barrier strength. While the exact data on permeability of the human intestine are sparse, there is a consensus that the human intestine presents a relatively weak barrier with TEER values reported in the range of ~100 Ohms×cm^2^ [53]. Almost all reports of Caco-2 monolayers, regardless of the method of TEER measurement, report far greater, 500 to over 2000 Ohms×cm^2^, values [53]. Co-culture of Caco-2 cells with other cell types typically leads to lower TEER [54,55]. Similarly, TEER values in MPS have also been reported as approaching 1000 Ohms×cm^2^ [32,56]. Thus, our finding that J2 and D109 cells typically had a larger TEER than Caco-2 cells may not indicate a more physiologically relevant system. While higher TEER would indicate restrictions on passive diffusion of the small molecules, we did not observe this to be the case with 100 min exposures in the comparisons of *P*_app_ and *F*_abs_ for caffeine, propranolol, and indomethacin. In fact, the EpiIntestinal^TM^ model’s data were most different from the known human near-complete absorption of these compounds, even though it had the lowest TEER that was also closer to the assumed human intestine TEER. Also, many laboratories use TEER as a quality control check, for example, the Caco-2 MPS study of 19 small molecules [24] required TEER to be >300 Ohms×cm^2^ before a sample was deemed useable. Thus, we conclude that taking TEER at the face value as an indicator of physiological relevance in isolation from other data may not be the most appropriate benchmark for complex in vitro systems.

Collectively, our study lays the foundation for several future research directions. First, optimization of MPS conditions will be necessary to reduce variability in the readouts. Further engineering may be needed to enable stable enteroid-derived cultures under flow, which could unlock the full potential of these models in dynamic systems. Second, future studies will need to test a larger number of compounds and be expanded to other drug classes, particularly those requiring xenobiotic metabolism for absorption and first-pass metabolism. Testing of a broader range of compounds, including biologics, or efflux substrates will clarify how these models perform under pharmacologically challenging scenarios. Third, incorporating immune and microbiota components of the gut into studies of absorption and metabolism may be desired. This could be achieved through the incorporation of additional cell types (immune, endothelial, and microbiota) into these systems and enhance their relevance beyond studies inflammation, host–microbe interactions, and disease modeling [57]. Still, additional research is needed to understand in which context of use such complexity might bring a benefit in predicting human absorption and define the cost–benefit ratio of these systems. Finally, additional work on standardization and validation is needed to promote the use of enteroid and MPS models across labs to facilitate regulatory acceptance and industry uptake.

In conclusion, this study underscores the value of enteroid-based models and supports their broader adoption in intestinal permeability and drug absorption research. While MPS hold promise, standard assays with well-characterized human-derived cells currently may offer a sensible balance of physiological relevance, reproducibility, and practical utility. Through a direct comparison of model systems and integration with in silico tools, we contribute new knowledge toward the optimization and future standardization of in vitro gut models for pharmaceutical and toxicological applications.

## Data Availability

The data presented in this study are openly available and can be found on the EveAnalytics Database using the links for each study that are listed in Table S1.

## Supplementary Materials

The following supporting information can be downloaded at: https://www.mdpi.com/article/10.3390/bioengineering12080809/s1. Table S1: Hyperlinks to all raw data and study protocols. Individual studies are matched to the figures in which the data were presented, Table S2: Data collected from reference documents for range of *P*_app_ values for caffeine, propranolol, and indomethacin.
