# Supplementary material for: Human Small Intestinal Tissue Models to Assess Barrier Permeability: Comparative Analysis of Caco-2 Cells, Jejunal and Duodenal Enteroid-Derived Cells, and EpiIntestinalTM Tissues in Membrane-Based Cultures with and Without Flow
Source: Bioengineering (Basel). 2025 Jul 28;12(8):809. doi: 10.3390/bioengineering12080809 (PMC12384003; doi:10.3390/bioengineering12080809)
Supplement: Supplementary file 1 [file bioengineering-12-00809-s001.zip › bioengineering-3760551-supplementary.pdf]

Table S1. Hyperlinks to all raw data and study protocols. Individual studies are matched to the figures in which the data were presented.

Figures	Cell type	Model	Study Name in Database	URLs for full data access
2, 3, 4, 5, 6, 7	D109	Static vs flow	TEX-VAL-CC-2021-11-17-CBE16 D109 Enteroid drug permeability-Transwell/CNBIO static vs flow	https://eve.eveanalytics.com/assays/assaystudy/745/
2, 3, 4, 5, 6, 7	J2	Static vs flow	TEX-VAL-CC-2021-11-12-CBE15 J2 Enteroid drug permeability-Transwell/CNBIO static vs flow	https://eve.eveanalytics.com/assays/assaystudy/799/
2, 3, 4, 5, 6, 7	J2	Static vs flow	TEX-VAL-CC-2021-12-09-CBE17 J2 Enteroid drug permeability-Transwell/CNBIO static vs flow	https://eve.eveanalytics.com/assays/assaystudy/802/
2, 3, 4, 5, 6, 7	D109	Static vs flow	TEX-VAL-CC-2022-01-09-CBE20 D109 Enteroid drug permeability-Transwell/CNBIO static vs flow	https://eve.eveanalytics.com/assays/assaystudy/803/
2, 3, 4, 5, 6, 7	J2	Static vs flow	TEX-VAL-CC-2022-01-12-CBE21 J2 Enteroid drug permeability-Transwell/CNBIO static vs flow	https://eve.eveanalytics.com/assays/assaystudy/804/
2, 3, 4, 5, 6, 7	J2	Static vs flow	TEX-VAL-CC-2022-01-30-CBE23 J2 Enteroid drug permeability-Transwell/CNBIO static vs flow	https://eve.eveanalytics.com/assays/assaystudy/805/
2, 3, 5, 6, 7	Caco2	Static vs flow	TEX-VAL-CC-2022-01-13-TC13 caco2 drug permeability-Transwell/CNBIO static vs flow	https://eve.eveanalytics.com/assays/assaystudy/806/
2, 3, 5, 6, 7	Caco-2	Static vs flow	EX-VAL-CC-2022-01-20-TC14 caco2 drug permeability-Transwell/CNBIO static vs flow	https://eve.eveanalytics.com/assays/assaystudy/808/
2, 3, 5, 6, 7	Caco-2	Static vs flow	TEX-VAL-CC-2022-02-11-TC15 caco2 drug permeability-Transwell/CNBIO static vs flow	https://eve.eveanalytics.com/assays/assaystudy/809/
2, 3, 5, 6, 7	MatTek	Static	TEX-VAL-CC-2022-07-26-MAT01 Mattek Epi-Intestinal	https://eve.eveanalytics.com/assays/assaystudy/1029/

Table S2. Data collected from reference documents for range of Papp values for caffeine, propranolol, and indomethacin.

Chemical	P_{app} range (*10 $^{-6}$ cm/s^2)	Collected From	DOI
Indomethacin	1.12 ± 0.08	Khan et al., 2011	https://doi.org/10.3109/1061186X.2010.531730
Caffeine	44.29 ± 5.12	Kus et al., 2023	DOI: 10.3390/pharmaceutics15112523
Propranolol	30.76 ± 1.91	Kus et al., 2023	DOI: 10.3390/pharmaceutics15112523
Propranolol	49.5 ±1.2	Teksin et al., 2010	DOI: 10.1208/s12248-010-9176-2
Indomethacin	38.4 ±2.2	Teksin et al., 2010	DOI: 10.1208/s12248-010-9176-2
Caffeine	30.8-50.5	O'Hagan and Kell, 2015	DOI: 10.7717/peerj.1405
Propranolol	8-232	O'Hagan and Kell, 2015	DOI: 10.7717/peerj.1405
Indomethacin	20-109	O'Hagan and Kell, 2015	DOI: 10.7717/peerj.1405
